# The Book World of Medicine and Science

**Published:** 1902-08-30

**Authors:** 


### The Book World of Medicine and Science.

The Earth in Relation to the Preservation and Destruction of Contagia. By George Vivian Poore, M.D.(Lond.), F.R.C.P. (London: Longmans, Green and Co. 1902. Price 5s. net.)

We have in this volume the Milroy Lectures delivered by the author at the Royal College of Physicians in 1899, together with certain addresses to the Royal Medical and Chirurgical Society, and other papers on sanitary subjects, almost all of which we have mentioned with appreciation at the time of their appearance. There can be no doubt that Dr. Poore has done well to issue these addresses in their present form since, taken collectively, they present with a vigour and a wealth of illustration which is not to be found elsewhere certain views which ought to be constantly kept in mind by those who have to do with such matters as drainage and water supply. That what Dr. Poore says in regard to the evils which so often result from the use of water as a means of removing excreta is true, we have no doubt whatever, and that to put to such vile uses so valuable a commodity is in many cases a wasteful proceeding, we may readily admit. All the same, while granting the truth of what he says, and while thoroughly agreeing with him that all great cities ought to have been built upon a far more open plan than they have been, we cannot bring ourselves to condemn off-hand the general plan of water carriage which has been adopted all over the world as a means of conservancy for towns and cities. Our towns exist, and we have to make the best of them, and for that purpose tap-water and public sewers stand at present as the best that can be done by municipal socialism in dealing with two of the most essential factors in healthy living.

In country districts things are different, and although, even now, we are not convinced that economy of labour does not lie on the side of taps and sewers, and although we are quite certain that if we were going to build a suburban villa our first inquiry would be whether the water was "laid on" and whether the sewers were "taken over," we are equally clear that in really rural districts the ingenious plans described by Dr. Poore for disposing of domestic, sewage and for obtaining a good supply of safe water are worthy of the most careful consideration. It is quite certain that in many country districts in which the sanitary authorities now wink at insanitary conditions in regard to cottages—conditions which are allowed to continue because it is imagined that there is no alternative except the expensive one of "company water" and "district sewerage"—everything could be put right and every reasonable requirement could be fulfilled by the adoption of plans so ably urged by Professor Vivian Poore. We cordially recommend this book to all country sanitarians.

Massage in Recent Fractures; Internal Derangements of the Knee-joint and the Management of Stiff Joints. By Sir William H. Bennett, K.C.V.O., F.R.C.S., Senior Surgeon to St. George's Hospital. (London: Longmans, Green & Co. 1902. With 17 Illustrations. Price 6s.)

Perhaps none of the revolutions in medical science during recent years are so striking as are those relating to the treatment of injuries. A few years ago rest was the sheet anchor of surgery; now massage and active and passive movements are begun by many surgeons at the earliest possible date after all kinds of fractures, sprains and contusions. The author of the present work claims to be the first to use methodically this system of treatment in England, and the results he has obtained must be described as essentially most satisfactory. Massage is begun at once after a fracture, passive movements of the joints near the injury in a few days, and active movements a little later. In cases of fractures of the patella, the author states "it is remarkable how little, if any, stretching of the union occurs in cases treated in this manner," although he is careful to add that he has no "wish to substitute in a general way treatment by massage and manipulation for the operative measures now so commonly employed in fractures of the olecranon and patella." Sir William Bennett also demonstrates the great aid massage is in the reduction of dislocations, in derangements of the knee-joint, and in various lesions and stiff joints. The work consists of four lectures, carefully edited, and is well illustrated by photographs and skiagrams.

Facts about Flogging. By Joseph Collinson. (London: William Reeves, 83 Charing Cross Road, W.C. Price 6d.)

The main contention of Mr. Collinson's pamphlet is that flogging does not frighten a criminal into a decent life, but by brutalising him makes him more inclined to evil than before. To prove this he quotes various instances of criminals who have been flogged more than once, after different convictions. "The criminal statistics of the Home Office," he says, "teem with cases of men who have been flogged more than once—two or three times many of them. Ned Wright, the Hoxton burglar, was flogged six times." The argument is that flogging is no deterrent. The brochure, however, suffers from the inevitable defects of an *ex parte* statement.

Some Results of the Nordrach Treatment of Consumption in Ireland. By P. S. Hichens, M.A., M.B., B.C.L. (Dublin: T. Falconer. 1901. Pp. 11, paper. Printed for the Author.)

It is pleasant to learn, from the pages of Dr. Hichens's pamphlet, that the methods of Nordrach, so successful in England, are not less successful in the humid atmosphere of the Emerald Isle. Dr. Hichens has for a year past treated at Rossclare, on the shores of Lough Erne, cases of pulmonary phthisis by insisting, to quote his own words, on three simple things—fresh air at all times, superabundance of food, and exercise graduated according to the state of the body temperature. The results have been most encouraging, and it is instructive to gather, moreover, that appropriate drugs are usefully employed, that trained nursing is provided, and that the conditions of life at Rossclare generally are those attainable by most of the invalids after recovery. Dr. Hichens has increased the value of the illustrative reports given by him by clear and precise statements of the physical signs presented by his patients, both on admission and on recovery.

